# Influence of Structural Parameters on the Performance of an Asymmetric Rhombus Micromixer with Baffles

**DOI:** 10.3390/mi14030545

**Published:** 2023-02-26

**Authors:** Jiacheng Nai, Feng Zhang, Peng Dong, Ting Fu, Anle Ge, Shuang Xu, Yanqiao Pan

**Affiliations:** 1 Hubei Key Laboratory of Mechanical Transmission and Manufacturing Engineering, Wuhan University of Science and Technology, Wuhan 430081, China; 2 Key Laboratory of Metallurgical Equipment and Control Technology, Ministry of Education, Wuhan University of Science and Technology, Wuhan 430081, China; 3 Precision Manufacturing Institute, Wuhan University of Science and Technology, Wuhan 430081, China; 4 Single-Cell Center, Qingdao Institute of Bioenergy and Bioprocess Technology, Chinese Academy of Sciences, Qingdao 266101, China

**Keywords:** micromixer, structure optimization, baffle, mixing index, numerical simulation

## Abstract

As an important part of lab-on-a-chip and micro-total analysis systems, micromixers have a wide range of applications in biochemical analysis, pharmaceutical preparation and material synthesis. In the work, a novel rhombic separation and recombination micromixer with baffles was presented to further improve the performance of the micromixer and study the effect of multiple structural parameters on mixing. The effects of the rhombic angle, the width ratio of sub-channel and the size and relative positions of baffles on the mixing index were studied numerically at different Reynolds numbers (*Re*), and the sensitivity of the mixing index to various structures was also investigated. The results showed that the mixing index increased with the subchannel’s width ratio and slowly decreased after reaching the peak value in the range of *Re* from 0.1 to 60. The maximum mixing index appeared when the width ratio was 6.5. The pressure drops in the microchannel were proportional to the width ratio. The mixing effect can be further improved by adding baffle structure to asymmetric rhombus micromixer, and more baffle quantity and larger baffle height were beneficial to the improvement of the mixing index. The research results can provide reference and new ideas for the structure design of passive micromixers.

## 1. Introduction

Microfluidic chip devices are used to analyze the chemical reaction process in the fields of biology, medicine, chemical analysis and environmental monitoring and can integrate the basic operation units, such as injection, mixing, reaction, washing, separation and detection into a micro-nano scale chip, which has the characteristics of compact size, flexible combination, scale integration, fast detection, high sensitivity and low cost [1,2].

As an important component of microfluidic technology, micromixers are often used for the pretreatment of experimental samples in chemical and biological analysis to improve the detection efficiency; they can be divided into passive and active micromixers depending on whether an external energy source is required [3,4,5,6,7,8,9]. Most active micromixers have complex structures and manufacturing processes, while passive micromixers have a wide application prospect in microfluidic technology due to their simple structure and the ability to effectively improve mixing efficiency by optimizing their internal structure [10]. However, the magnitude of *Re* is limited by the channel size of micro or nano scale, resulting in laminar flow, which makes the mixing difficult. Therefore, changing the flow state of the fluid by optimizing the channel structure of the micromixer has become the research focus of efficient mixing [11,12,13,14,15].

So far, researchers have mainly focused on studies of passive micromixers with T-type [16,17,18,19,20,21], Z-type [22,23] and Y-type [24,25,26] channel structures. They found that mixing in microchannels is mainly dependent on molecular diffusion and confirmed that passive micromixer optimization can improve mixing efficiency by creating a flow state for extruded or stretched fluid by placing an effective channel structure within the microchannel [5]. Zhang et al. [27] reported a micro-mixer with multiple rhomboid channels and studied the influence of the rhomboid angle and the rhomboid channel width ratio on the mixing performance through numerical simulations. Chung et al. [28] designed a branch rhombic micromixer and found that increasing the contact interface area and chaotic convective eddy current and reducing the flow resistance of the branch channel could significantly improve the mixing efficiency through both numerical and experimental methods. Cortes-Quiroz et al. [29] proposed a 3D T-shaped mixer and found that design characteristics had a strong influence on the flow characteristics in the microchannels, and effective structural design can lead to the rapid formation of eddy current structure, thus, enhancing the mixing. Liu et al. [30] proposed a 3D cross-linked double-helix micromixer, studied the fast mixing process under a low *Re* number by using a numerical method, and found that mixing units such as split recombination, chaotic advection and flow impact could improve mixing efficiency in the microchannel. Juraeva et al. [31] proposed a passive micromixer stacked with eight mixing cells, in which each mixing cell was provided with a baffle to make the fluid disturbance in the channel stronger and the mixing efficiency higher. Xia et al. [32] proposed a chaotic mixer with a gap and a baffle structure. By this, a multidimensional eddy current inside the micromixer was generated, which promoted the molecular diffusion and improved the mixing efficiency. Zhang et al. [33] proposed a passive micromixer with a repeating cube mixing unit and studied the influence of non-equilibrium vortices and cell connection forms on mixing performance. Ansari et al. [34] proposed a separation and recombination micromixer with circular and rhomboidal subchannels that created unbalanced collisions by changing the width of the separation channels. These studies revealed that the design of channels or baffles can promote fluid mixing in channels. Subchannels with different shapes can split and recombine the fluid and collide at the junction of channels to improve the mixing effect. Placing baffles in the channel creates eddies that enhance fluid perturbation and stretch the fluid, increasing the contact surface and facilitating mixing. At present, there are sufficient research bases for the use of mixers for each channel structure; however, there are not many reports on the combined effects of multiple structural parameters (angle of subchannel, width ratio, baffle size and position) on the mixer simultaneously.

In order to comprehend the comprehensive influence of multiple structural parameters on the mixing effect, a novel micromixer with baffle and asymmetric channel structure is proposed based on the common rhomboid channel mixer. The flow field and concentration distribution in the micromixer are analyzed through numerical simulation after the visual flow velocity measurement experiment. The effects of the splitting and closing angle, the width ratio and the size and location of baffles on the comprehensive performance of the micromixer were also discussed through the numerical results in the range of *Re* = 0.1, 1, 10, 20, 40 and 60, thus, investigating the sensitivity of the mixing performance to each structure.

## 2. Geometry of the Micromixer

**Figure 1:**
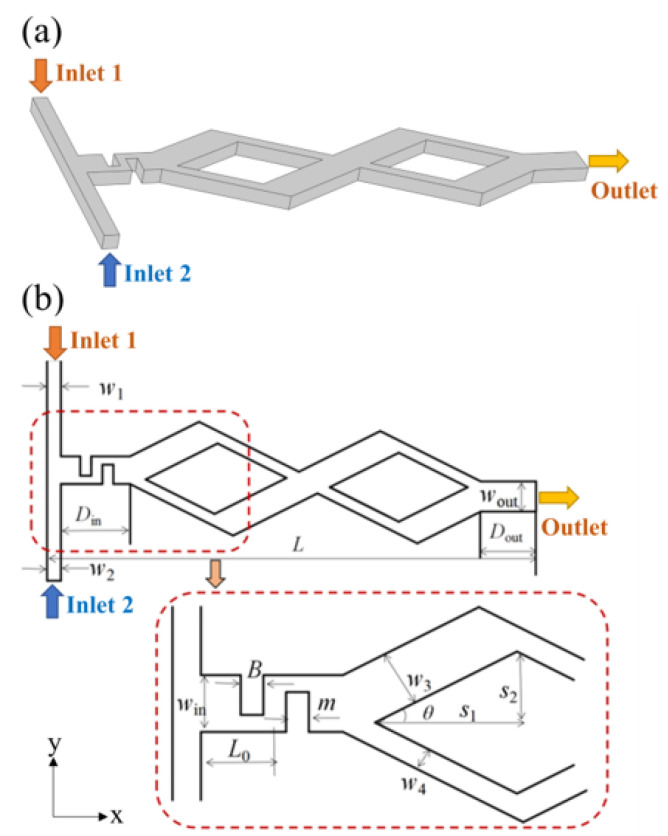
Sketch of the micromixer. (**a**) 3D view; (**b**) Cross section of micromixer.

The sketch of the asymmetric rhomboidal chaotic micromixer with a baffle structure is shown in Figure 1a, which consists of a T-shaped inlet channel, a baffle area and a rhomboidal optimized channel. Fluid A and B enter through Inlet 1 and Inlet 2, respectively, and exit through the Outlet. Figure 1b shows the horizontal section of the channel, and the micromixer sizes are listed in Table 1.

## 3. Numerical Model and Verification

### 3.1. Numerical Model

In the numerical model, the fluid A and B in the microchannel are assumed to be a steady state incompressible Newtonian fluid, and the influence of gravity is ignored, which satisfies the continuity equation and the Navier–Stokes equation, which are:

(1) $$\nabla \cdot \vec{v}=0,$$

(2) $$\rho \left(\vec{v}\cdot \nabla \right)\vec{v}=-\nabla p+\mu \nabla^{2}\vec{v},$$

where $\vec{v}$ is the velocity vector of the fluid, ρ is the density of the fluid, p is the pressure, and μ is the dynamic viscosity coefficient of the fluid. The model ρ = 998 kg/m^3^, μ = 0.001 Pa·s.

In order to solve the boundary conditions of the flow field, the non-slip boundary conditions are set at the channel walls. Inlet 1 and Inlet 2 are set as velocity boundary conditions, respectively, and the initial velocity at each inlet is 0.001 m/s. The pressure boundary condition is set at the Outlet, and the pressure is set to 0.

The concentration transport in the channel satisfies the convective diffusion equation of concentration, which is:

(3) $$\frac{\partial C}{\partial t}+\vec{v}\cdot \nabla C=D\nabla^{2}C,$$

where *C* is the concentration of the solute to be mixed, and *D* = 1 × 10^−11^ m^2^/s is the diffusion coefficient of the solute. The concentration boundary conditions are set at the channel entrance, the concentration $C_{1in}=1$ is set at the Inlet 1 boundary, and the concentration $C_{2in}=0$ is set at the Inlet 2 boundary. The concentration boundary condition at the outlet of the channel is:

(4) $$\vec{n}\cdot (-D\nabla C)=0,$$

where $\vec{n}$ is the external normal unit vector. The calculation formula for evaluating the mixing index on the section at the exit of the channel is as follows [35]:

(5) $$\eta =\left[1-\frac{\iint_{S}\left|C-C_{\infty }\right|dydz}{\iint_{S}\left|C_{0}-C_{\infty }\right|dydz}\right]\times 100\%,$$

where C is the concentration distribution on the exit section, the concentration when completely mixed is $C_{\infty }$ = 0.5, and $C_{0}$ = 0 is the concentration when the mixture is unmixed. The value of η is 0 < η < 1; η = 0 indicates unmixed state, and η = 1 indicates fully mixed state.

The *Re* is also an essential parameter, defined as:

(6) $$Re=\frac{\rho vD_{h}}{\mu },$$

(7) $$D_{h}=\frac{4A}{P},$$

where v is the mean velocity of the fluid, the velocity of two inlets is 0.01 m/s. $D_{h}$ represents the hydraulic diameter of the channel. A denotes the cross-section area of the channel, *P* represents the perimeter of the channel section.

**Figure 2:**
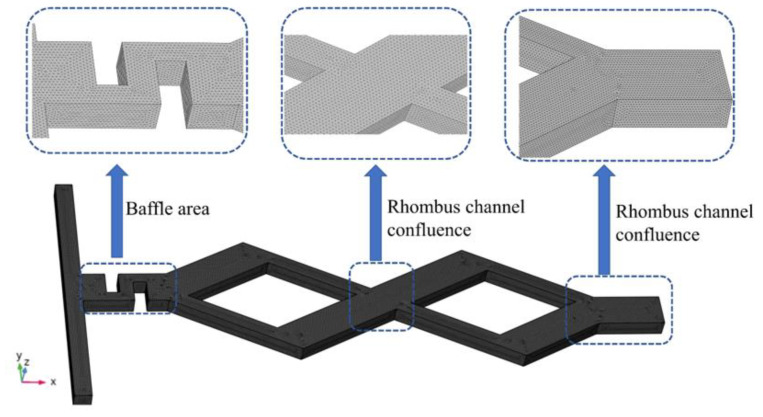
Computational mesh.

The control Equations (1) and (2) are solved numerically to obtain the flow field in the microchannel by the finite element software COMSOL Multiphysics 6.0. Then, the flow velocity is substituted into Equation (3) to solve for the concentration field. The computational mesh is shown in Figure 2, the discrete 3D model of triangular cells with Lagrangian quadratic functions is established, and the physical governing equations are numerically calculated using a finite element method.

The grid convergence index (GCI) was calculated to ensure the accuracy of the calculation results [36], and the absolute tolerance was specified to be 0.001 as the convergence criterion. The expression of GCI is shown as follows:

(8) $$GCI=F_{s}\frac{\left|\varepsilon \right|}{r^{p}-1},$$

(9) $$\varepsilon =\frac{f_{coarse}-f_{fine}}{f_{fine}},$$

where $F_{s}$, p and r are the safety factor, the order of accuracy of the numerical method, and grid refinement ratio, respectively. $f_{coarse}$ and $f_{fine}$ are the numerical results resulting from the coarse and fine meshes, respectively. $F_{s}$ takes the value of 3 as recommended in [37]. At *Re* = 1, the mixing index is used as the criterion. For the number of grids of 10 w, 25 w and 40 w, the value of GCI was calculated, and the results are shown in Table 2. The GCI of the mixing index is calculated as 2.437%. Therefore, the grid number of 25 w is chosen to perform the numerical solution.

### 3.2. Experimental Verification

**Figure 3:**
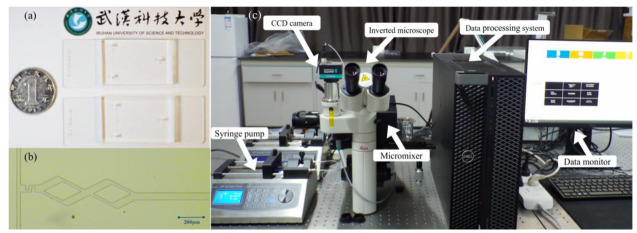
The photographs of the micromixer and experimental platform. (**a**) Photograph; (**b**) Photomicrograph; (**c**) Mirco-PIV. experimental platform.

A microfluidic chip (fabricated by the Institute of Bioenergy and Processes, Qingdao, Chinese Academy of Sciences) was fabricated by etching polydimethylsiloxane (PDMS) with microchannels and bonding to a glass substrate to verify the accuracy of the numerical results. The photographs of the micromixer and experimental platform are shown in Figure 3. As shown in Figure 3c, a Mirco particle image velocimetry (Mirco-PIV) experimental platform (Dantec Dynamics, Skovlunde, Denmark) was used to obtain the fluid velocity vector in the microchannel, and it will be compared with the numerical simulation results.

**Figure 4:**
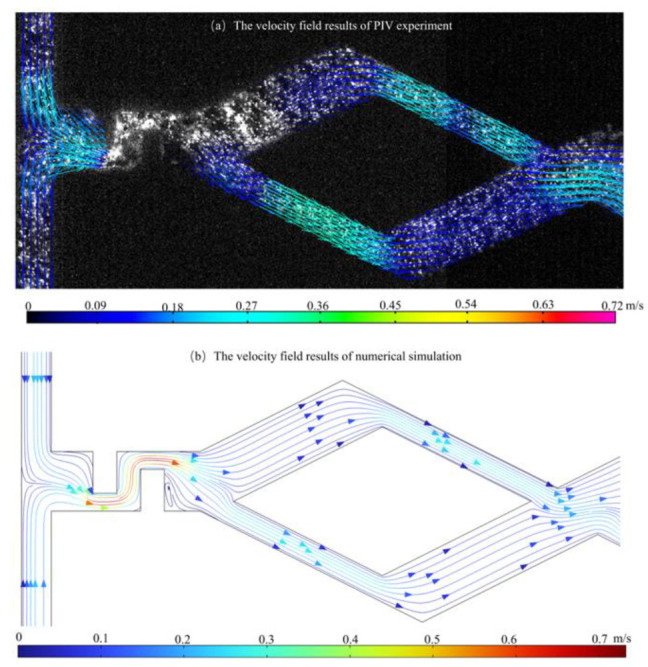
Fluid flow state in micromixer. (**a**) Experimental results; (**b**) Numerical results.

Deionized water containing 1 μm diameter tracer particles (FluoroSpheres, Thermo Fisher Scientific, Waltham, MA, USA) was injected into Inlet 1 and Inlet 2 by two microinjection pumps (SP02-1B dual-channel extraction perfusion type, Chuangdi Electronic Technology, Baoding, China). Figure 4a,b shows the experimental results of the fluid velocity field collected by the Mirco-PIV experimental platform and the numerical results under the same experimental conditions, respectively. The experimental and numerical results of the maximum velocity in a wide channel are 0.0996 m/s and 0.106 m/s, respectively, with a relative error of 6.13%, and the maximum velocity in a narrow channel is 0.269 m/s and 0.2787 m/s, respectively, with an error of 3.48%.

**Figure 5:**
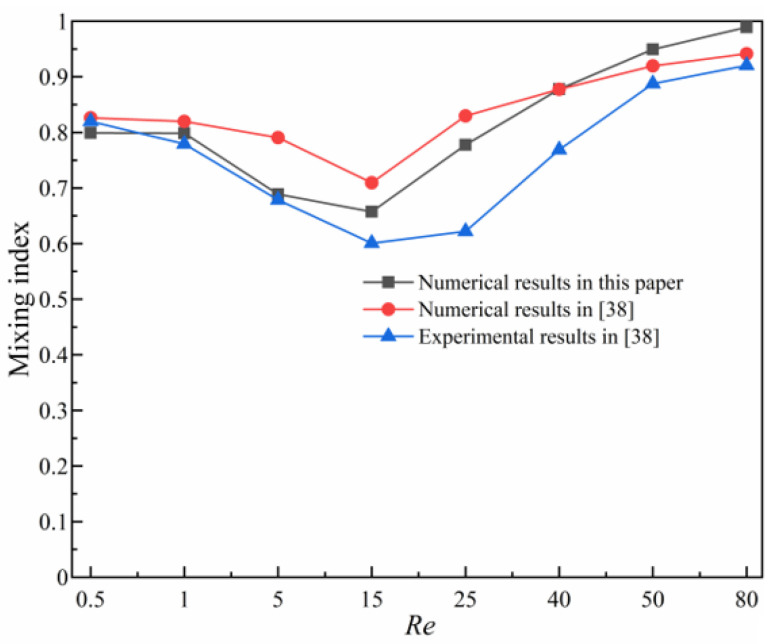
Comparison of numerical results of mixing index with results in [38].

Experimental results of the micromixer in reference [38] are used to validate the computational model and numerical results. A 3D micromixer model with the same structure in [38] is set up and the results of the concentration field in the mid-section at *Re* = 0.5, 1, 5, 15, 25, 40, 50, 80 are presented in Figure 5, which shows the numerical results of the mixing index obtained by the computational model in this paper, and the experimental and numerical results in [38]. As is shown in Figure 5, the results of the mixing index obtained from our computational model correspond well with results in [38].

## 4. Results

**Figure 6:**
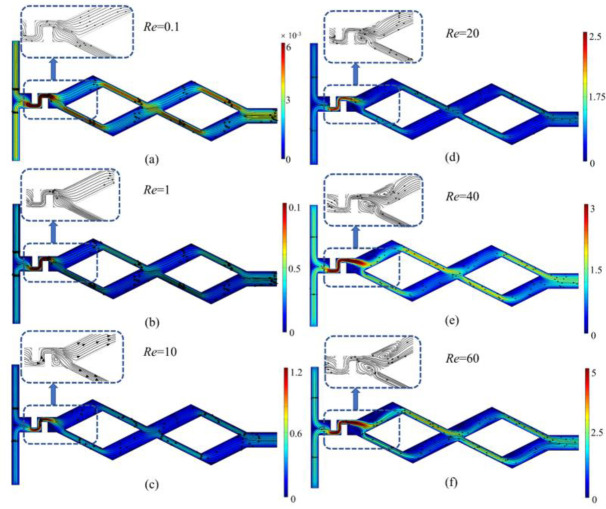
Velocity field distribution (m/s) of asymmetric rhombus micromixer with baffles at *Re* = 0.1, 1, 10, 20, 40 and 60. (**a**) *Re* = 0.1; (**b**) *Re* = 1; (**c**) *Re* = 10; (**d**) *Re* = 20; (**e**) *Re* = 40; (**f**) *Re* = 60.

The velocity field distribution of the asymmetric rhombus micromixer with baffles at *Re* = 0.1, 1, 10, 20, 40 and 60 is shown in Figure 6, where the colors represent the velocity magnitude, and the arrows represent the velocity direction. As shown in Figure 6a–f, the two fluids enter the microchannel from the inlet, respectively, and are squeezed and folded in the channel placed with baffles, and the eddy is gradually generated in the baffle area and increases with the *Re*.

**Figure 7:**
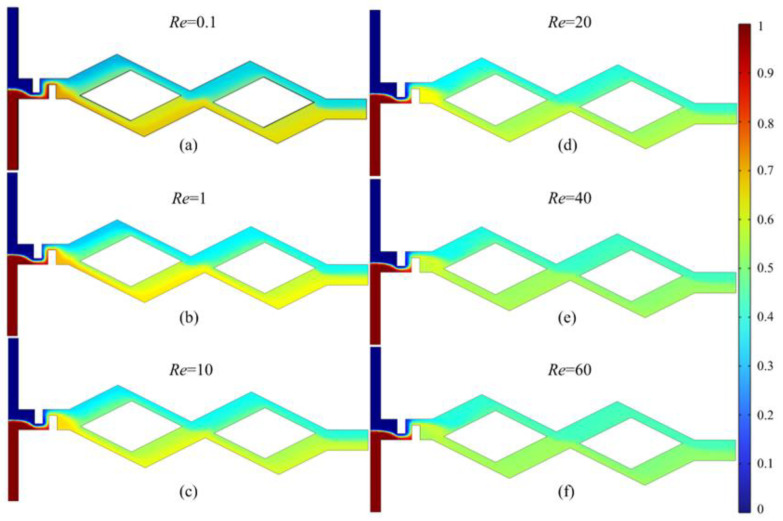
Concentration field distribution of asymmetric rhombus micromixer with baffles at *Re* = 0.1, 1, 10, 20, 40 and 60. (**a**) *Re* = 0.1; (**b**) *Re* = 1; (**c**) *Re* = 10; (**d**) *Re* = 20; (**e**) *Re* = 40; (**f**) *Re* = 60.

Figure 7 shows the concentration field distribution at *Re* = 0.1, 1, 10, 20, 40 and 60. The initial mixing of the fluids happens after the folding and squeezing of the baffle. Subsequently, the fluids flow through two rhombic channel units and are further mixed after unbalanced collisions at the confluence of the rhombic channels. It can be found in Figure 7 that the mixing effect becomes more intense with the increase in *Re*.

Assuming the characteristic area and viscosity of the fluid remained unchanged. When *Re* = 0.1 and 1, the mixing of the fluid mainly depends on diffusion. Lower flow velocity gives the fluid more time to diffuse, so the mixing index of the solution decreases with the increase in flow velocity at lower *Re*. When *Re* = 10, 20, 40 and 60, the influence of natural diffusion on the mixing efficiency gradually reduces, and the fluid convection plays a dominant role.

**Figure 8:**
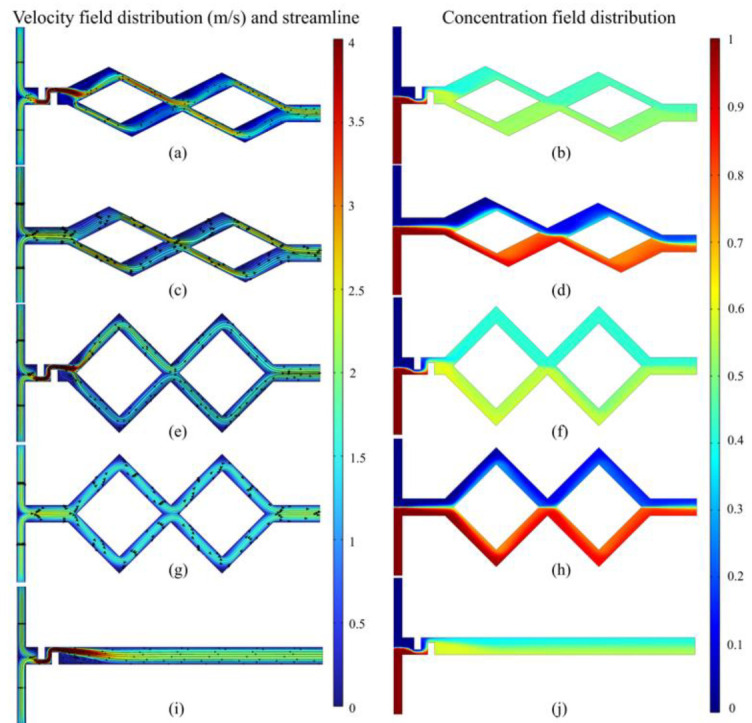
Velocity field distribution (m/s) and concentration field distribution of five micromixers at *Re* = 60. (**a**,**b**) Asymmetric rhombus micromixer with baffles; (**c**,**d**) Asymmetric rhombus micromixer without baffles; (**e**,**f**) Symmetric rhombus micromixer with baffles; (**g**,**h**) Symmetric rhombus micromixer without baffles; (**i**,**j**) Micromixer only with baffles.

Figure 8 indicates the velocity field distribution and concentration field distribution of five micromixers at *Re* = 60. As shown in Figure 8a–d, after the fluid is separated in the first rhombic channel, the wide and narrow structure of the sub-channel in the asymmetric rhombic micromixer results in the unbalanced collision of two fluids with different masses at the confluence of the rhombic channel under the action of inertial forces. The high velocity flow in the narrow channel and the low velocity flow in the wide channel converge and collide; the momentum conversion occurs. Therefore, the contact surface between the different component fluids becomes blurred, which increases the contact area between the two fluids, thus, improving the mixing efficiency. In the symmetric rhombic micromixer (Figure 8e–h), the overall velocity distribution of the fluid is evenly distributed, and the momentum conversion effect is weaker at the confluence of the rhombic channels. The two fluids remained in a stratified flow condition after the collision at the confluence of the rhombus channel, and thus, the fluid mixing effect is not satisfactory.

As presented in Figure 8c,d,g,h, the velocity is evenly distributed in the inlet area, and the fluid flow is stratified. The fluid mass mixing basically depends on the molecular diffusion in the channel. In contrast, the solute mixing effect is obvious after adding the baffle structure in Figure 8a,b,e,f; eddy currents are generated in the channel and the fluid flows are disturbed under the action of baffle structures, which promotes the initial mixing. As shown in Figure 8i,j, in the micromixer only with baffles, the fluid flows uniformly and stratifies in the channel after the folding and squeezing by the baffle; the mixing effect is similar to the symmetrical rhombic micromixer with baffles (Figure 8f).

**Figure 9:**
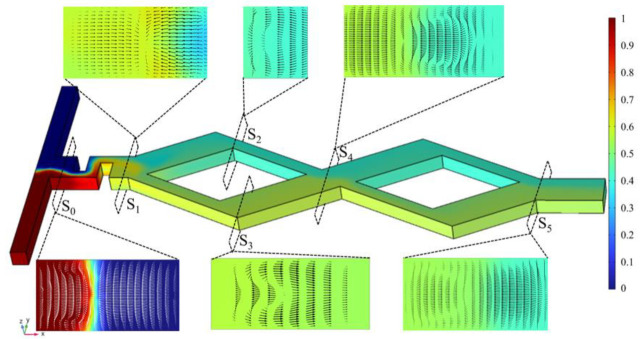
Concentration distribution and velocity vectors on different sections S_0~_S_5_ at *Re* = 60.

In order to further analyze the strengthening effect of the baffle and asymmetric rhombic channel on the mixing efficiency, six different sections from S_0_~S_5_ were selected in the y–z plane in the vertical flow direction, and the concentration distribution and velocity vector of the section were analyzed under the condition at *Re* = 60. As shown in Figure 9, the fluids converge at the T-shaped inlet S_0_, where there is an obvious partition interface between the two fluids; After squeezing by the baffle channel structure, the fluid flows out from the narrow slit and diffuses in the S_1_ section, and then separates at the rhombic wide and narrow branch; When the fluid flows through the S_2_ and S_3_ sections at the turning point of the rhombic channel, the secondary flow in the form of a dean eddy is generated in the plane that is perpendicular to the flow direction under the action of centrifugal force and, thus, further promotes fluid mixing; As the fluid flows through the sections of S_4_ and S_5_, fluids in the wide and narrow channel have an unbalanced collision at the confluence of the rhombic channel, which leads to the generation eddy currents on both sides of the channel. The fluid in the center of the channel is exchanged with the fluid at the edge, and hence, the mixing is carried out more completely.

**Figure 10:**
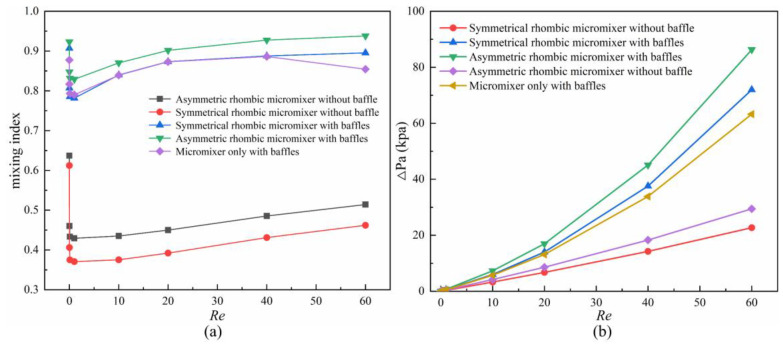
Comparison of five rhombic mixers. (**a**) Mixing index curves; (**b**) Pressure drop curves.

Figure 10 displays the mixing index curves and pressure drop curves of the five rhombic mixers when *Re* = 0.001, 0.1, 1, 10, 20, 40 and 60, θ = 26.5°, and the width ratio of the sub-channel is 2.5; the distance between the two baffles with a length of 70 μm and a width of 40 μm is 30 μm, and the baffle1 is placed 150 μm to the inlet. As shown in Figure 10a, when *Re* = 0.001 and 1, the mixing index of the five micromixers shows a decreasing trend with the increase in *Re*. The mixing index of asymmetric rhombic micromixer increases by 0.31 to 0.44 compared with the regular rhombus one (symmetrical rhombic micromixer without baffle). It is obvious that the mixing index of the micromixers with baffles has a significant increase compared to the ones without baffles, with an increase of 0.286~0.481. In order to investigate the effect of the symmetric rhombus and asymmetric rhombus on the mixing index, we added a micromixer only with baffles for comparison. It can be found in Figure 10a that there is no obvious difference in the mixing index between the micromixer only with baffles and the symmetrical rhombic micromixer with baffles, This can be explained by Figure 8f,j, which show that the fluid continues to flow in layers in the microchannel after flowing through the baffles. As shown in Figure 10b, the pressure drop increases approximately linearly with *Re*. The pressure drop in the micromixers with baffles is significantly higher than the micromixer without baffles, and the difference increases with the *Re*. The magnitude of the pressure drop shows a positive relationship with the mixing index.

## 5. Discussion

### 5.1. Effect of Rhombic Angle on Mixing Performance

Assuming that the long half-axis of the rhombus remains unchanged, the value of the separating angle *θ* was changed with the variation of the short half-axis length. The value of *cot θ* was taken as the research object to discuss the influence of the separating angle on the mixing performance. The overall length of the channel varies with the short half-axis length, thus, affecting the mixing time and mixing efficiency. Therefore, the mixing index of unit length under different *cot θ* is as the standard of mixing performance.

**Figure 11:**
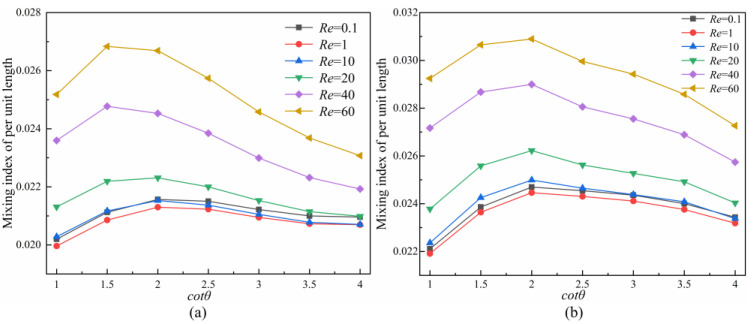
Effect of *cot θ* on the mixing index of per unit length at different *Re*. (**a**) Symmetric rhombus micromixer; (**b**) Asymmetric rhombus micromixer.

Figure 11 shows the mixing index per unit length with *cot θ* in symmetric and asymmetric rhombus micromixers and indicates that the mixing index increases with *cot θ* and decreases after reaching the peak. The mixing index changes subtly with the increase in *cot θ* when *Re* = 0.1, 1, 10 and 20, the fluid flow rate is low currently, and the fluid mixing to molecular diffusion is dominant, thus, the mixer structure has relatively less impact on the mixing efficiency. The mixing index of unit length is optimal at *cot θ* = 2. The mixing index changes significantly with the increase in *cot θ* when *Re* = 20, 40 and 60, and the fluid mixing at high *Re* is more significantly affected by the structure large.

The mixing index per unit length is the optimal at *cot θ* = 1.5 in the symmetric rhombus micromixer and at *cot θ* = 2 in the asymmetric rhombus micromixer, but there is little difference. For example, the unit mixing index is 2.157 × 10^−2^ (%/μm) at *Re* = 0.1 and *cot θ* = 2 in the symmetric rhombus micromixer; the unit mixing index is the least ideal at *cot θ* = 1, which is 2.02 × 10^−2^ (%/mm); the mixing index of per unit length is improved 1.067 times at this time.

**Figure 12:**
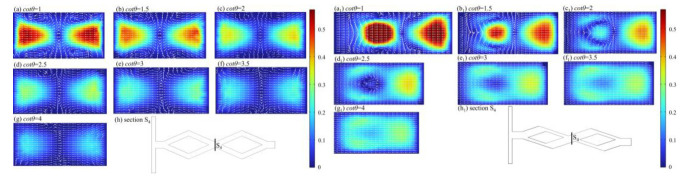
Velocity distribution (m/s) and vector of S_4_ section at *Re* = 60 under different *cot θ.* (**a**–**g**) Symmetric rhombus micromixer; (**h**) Section S_4_; (**a_1_**–**g_1_**) Asymmetric rhombus micromixer; (**h_1_**) Section S_4_.

Taking *Re* = 60 as an example, Figure 12 presents the velocity distribution and vector of S_4_ section under different *cot θ* in symmetric and asymmetric rhombus micromixers. As shown in Figure 12a–h, the results indicate that fluid vortices are generated at the top and bottom of the channel, which promote convective exchange between the middle fluid and the outer fluid, and thus, promoting fluid mixing. As *cot θ* increases, the gap between the velocity of the central region and the peripheral region gradually decreases, and thus, the collision effect of the two fluids at the confluence gradually diminishes. The vortices gradually move to both sides of the z-direction and gradually become less, and the convective diffusion effect between the fluids diminishes. The fluid velocity difference is most obvious at *cot θ* = 1. The fluid collision effect at the confluence is the optimal, but the channel length is also the longest; therefore, the unit mixing index when *cot θ* = 1 is less than that when *cot θ* = 1.5~3. Figure 12a_1_–h_1_ show that the cross-sectional velocity is asymmetrically distributed and eddies are generated at the top and bottom sides of the narrow channel when the unbalanced collision of fluids occurs. The eddies gradually move to the middle and decrease until disappear with the *cot θ*, and the velocity difference between the central region and the peripheral region on the section decreases gradually, indicating that the collision intensity of the two fluids at the junction decreases. For the rhombus mixer designed in this paper, *cot θ* = 1.5 is used as the size of the splitting angle.

### 5.2. Effect of Sub-Channel Width Ratio on Mixing Performance

In order to study the effect of the unbalanced collision caused by the asymmetric structure of the channel on the mixing intensity, the total width of the wide and narrow channels was set to 140 μm. The wide-to-narrow ratio (*w*_3_/*w*_4_) and the rhombic channel confluence (S_4_ cross-section) were used as the study objects, and the area of S_4_ cross-section varied with the magnitude of *w*_3_/*w*_4_.

**Figure 13:**
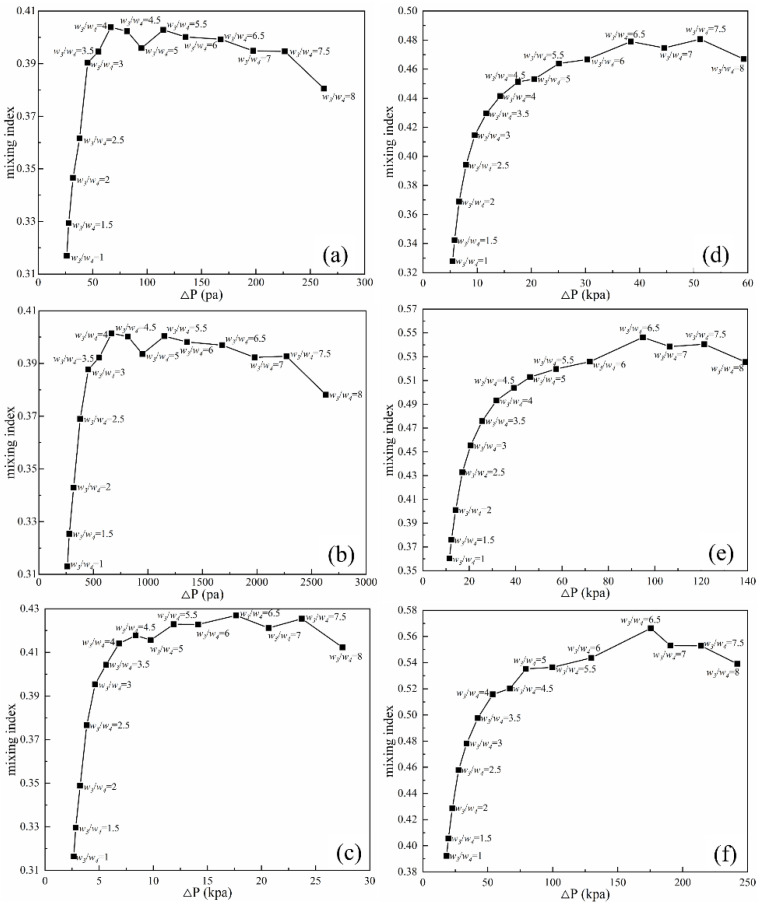
Mixing performance map. (**a**) *Re* = 0.1; (**b**) *Re* = 1; (**c**) *Re* = 10; (**d**) *Re* = 20; (**e**) *Re* = 40; (**f**) *Re* = 60.

Figure 13 shows the mixing performance map of the mixing index with pressure load at different *Re*, which indicates that the mixing index exhibits an increasing and then decreasing trend with the increase in *w*_3_/*w*_4_ at the same *Re*, and the pressure drop is rising. This is due to the significant difference in the quality and velocity of the narrow and wide channels. The intercourse between the two rhombus channels generates the inertia imbalance and interference of the two fluids (as shown in Figure 8a and Figure 9a). As *Re* increases, the maximum mixing index gradually moves toward a larger pressure drop (higher *w*_3_/*w*_4_). This means that the influence of *w*_3_/*w*_4_ becomes larger at higher *Re*. The maximum improvement of the mixing index is 0.1858 for different *w*_3_/*w*_4_ at *Re* = 40 compared with the micromixer without optimized width ratio (*w*_3_/*w*_4_ = 1). However, the pressure drop increases as the *w*_3_/*w*_4_ increases, and the channel pressure drop is improved 8.23 times. Considering that high pressure drop is unfavorable to the pipeline, the width ratio of 2.5 is chosen for the design of this paper.

**Figure 14:**
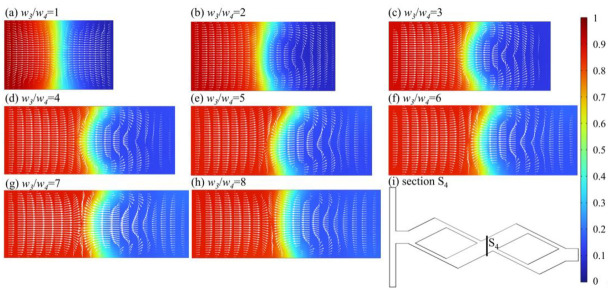
Concentration distribution and velocity vector of S_4_ cross section at *Re* = 60. (**a**) *w_3_/w_4_* = 1; (**b**) *w_3_/w_4_* = 2; (**c**) *w_3_/w_4_* = 3; (**d**) *w_3_/w_4_* = 4; (**e**) *w_3_/w_4_* = 5; (**f**) *w_3_/w_4_* = 6; (**g**) *w_3_/w_4_* = 7; (**h**) *w_3_/w_4_* = 8; (**i**) Section S_4_.

Figure 14 shows the concentration distribution and velocity vector of the S_4_ cross-section at *Re* = 60. The two streams of fluid (red and blue) balance collision in the middle of the section at ratio = 1, and the fluid interface is located in the middle of the section. As the ratio of *w*_3_/*w*_4_ increases, the eddy appears on the narrow channel, and the inertia imbalance of the fluid convergence is strengthened, resulting in the blur between the interface between the two fluids, the increase in the contact area, and the improvement of mixing efficiency. However, when the *w*_3_/*w*_4_ is overly large, the mass difference between the wide and narrow channel fluid is so large that there is no significant change in the S_4_ cross section by continuing to increase the *w*_3_/*w*_4_. Nevertheless, the pressure drop invariably rises with the *w*_3_/*w*_4_ increase. The larger pressure drop enables the fluid to leave the micromixer at a quicker flow rate, thus, reducing the mixing time in part by gradually reducing the mixing index.

### 5.3. Effect of Baffle Parameters on Mixing Performance

**Figure 15:**
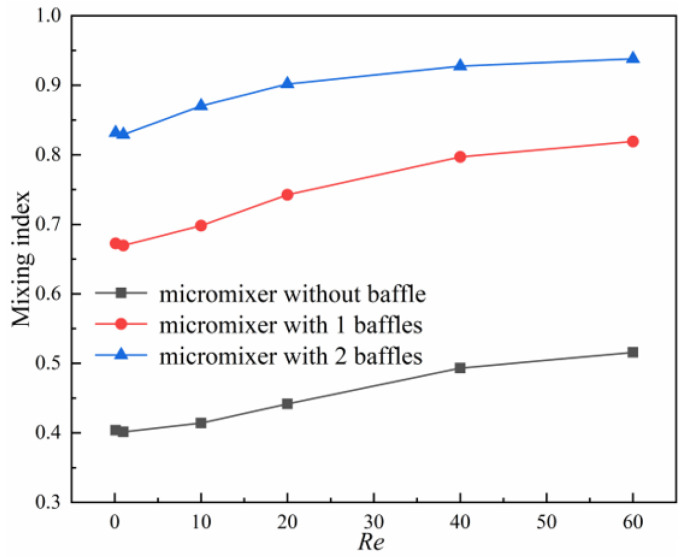
Effect of the number of baffles on mixing index.

In order to investigate the influence of baffles on mixing efficiency, the influence of the number of baffles on the mixing index at different *Re* is shown in Figure 15. Compared to the micromixer without baffles, the mixing index of the micromixer with two baffles increased by more than 0.42 in the range of *Re* = 0.1, 1, 10, 20, 40 and 60, and the mixing index of the micromixer with one baffle increased by more than 0.26, which means the baffle has a significant impact on the mixing efficiency.

#### 5.3.1. Effect of the Distance between the Baffle and the Inlet on Mixing Performance

**Figure 16:**
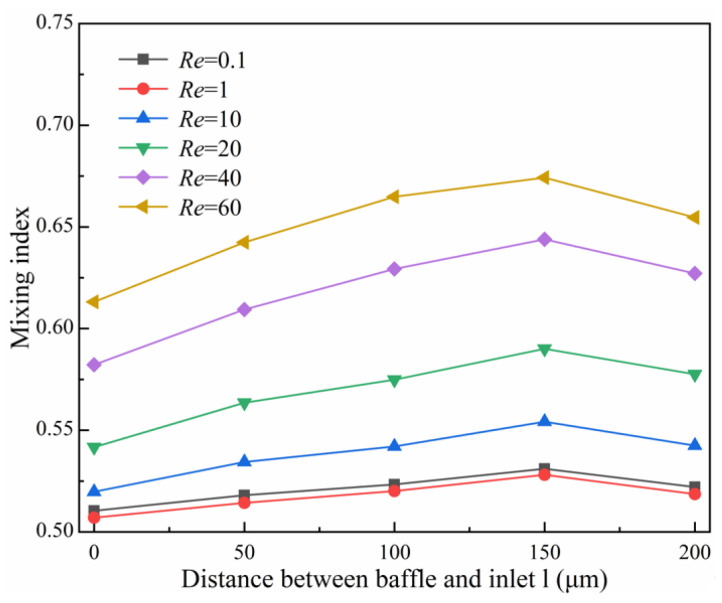
Effect of the distance between the baffle and inlet on mixing index.

**Figure 17:**
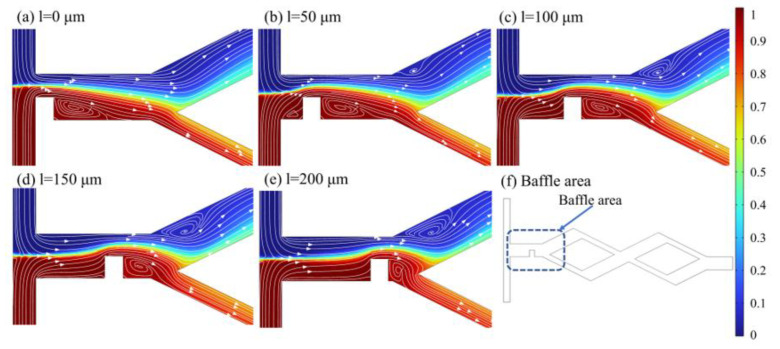
Concentration distribution and velocity streamline in the baffle area at *Re* = 60. (**a**) *l* = 0 μm; (**b**) *l* = 50 μm; (**c**) *l* = 100 μm; (**d**) *l* = 150 μm; (**e**) *l* = 200 μm; (**f**) Baffle area.

At *D_in_* = 250 μm and baffle height *h* = 50 μm, the mixing index varies with the distance *l* between the baffle and the inlet in the range of 0~200 μm for different *Re,* as shown in Figure 16. The results indicate that the mixing index shows a trend of increasing and then decreasing with the increase in *l*, and the mixing index is optimal when *l* = 150 μm. The maximum improvement in mixing index is 0.0617 at *l* = 150 μm compared to *l* = 0 μm when *Re* = 40. Taking *Re* = 60 as an example, Figure 17 shows the concentration distribution and velocity streamline at the baffle area, which indicates that an eddy current appears on the right side under the extrusion action of the baffle when *l* = 0 μm. The eddy current gradually appears in the rhombic wide channel with the gradual increase in *l*, thus, promoting more full mixing of fluids in the rhombic wide channel. As can be seen from Figure 17d, when *l* = 150 μm, the eddy size and strength generated in fluids A and B behind the baffle area are similar, resulting in an increase in the contact area of the two fluids and more conducive to mixing. As can be seen from Figure 17e, the reduction in the gap between the baffle and the rhombic channel when *l* = 200 μm has caused the vortex of the narrow channel entrance to become smaller and the wide channel to become larger, which has led to a decrease in the fluid contact area and reduced the mixing efficiency.

#### 5.3.2. Effect of Baffle Height on Mixing Performance

**Figure 18:**
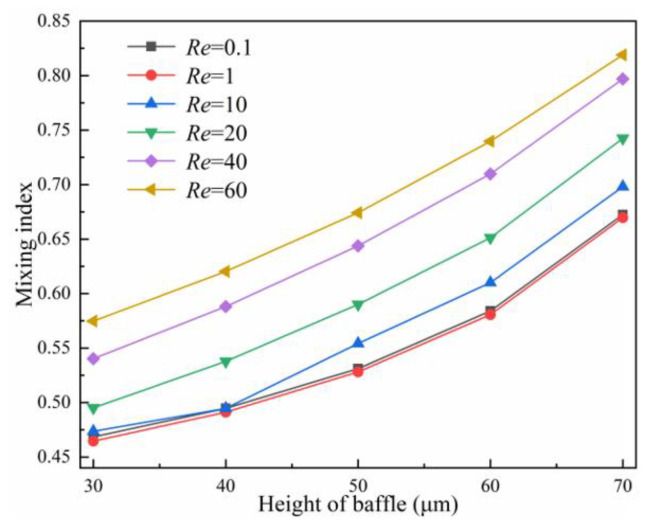
Effect of the height of baffle on mixing index.

**Figure 19:**
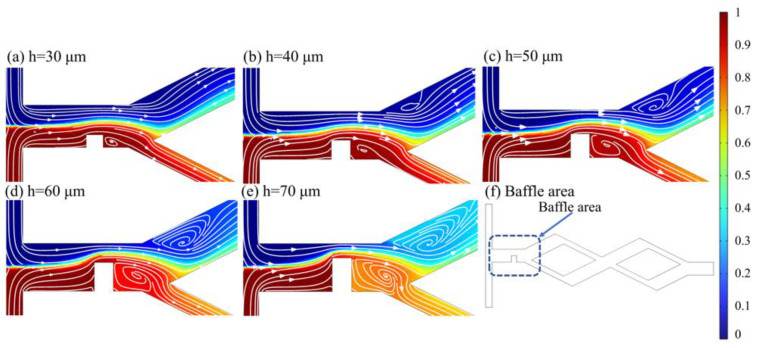
Concentration distribution and velocity streamline at the baffle area at *Re* = 60. (**a**) *h* = 30 μm; (**b**) *h* = 40 μm; (**c**) *h* = 50 μm; (**d**) *h* = 60 μm; (**e**) *h* = 70 μm; (**f**) Baffle area.

Setting *l* = 150 μm, the variation curves of the mixing index at different *Re* with baffle height *h* in the range of 30~70 μm are illustrated in Figure 18. The mixing index shows an upward trend with the increase in baffle height, and the mixing index is optimal at *h* = 70 μm. The maximum improvement in mixing index for baffle height *h* = 70 μm compared to *h* = 30 μm when *Re* = 40, with an increase of 0.2567. The concentration distribution and velocity streamline of the baffle area at *Re* = 60 are illustrated in Figure 19. The gap between the baffle and the side wall is smaller with the increase in *h*, and thus, the resistance and extrusion effect on the fluid are stronger. There is a larger eddy generated on the right side of the baffle and in the wide rhombic channel, thus, making the fluid mix more adequately. The concentration of the two streams changed obviously after flowing through the baffle structure when *h* = 70 μm (Figure 19e), and the mixing effect is obvious. However, overly high baffles create narrow slits in the channel, and the fluid bursts the channel if the width of the narrow slit is insufficient.

#### 5.3.3. Effect of Baffle Spacing on Mixing Performance

**Figure 20:**
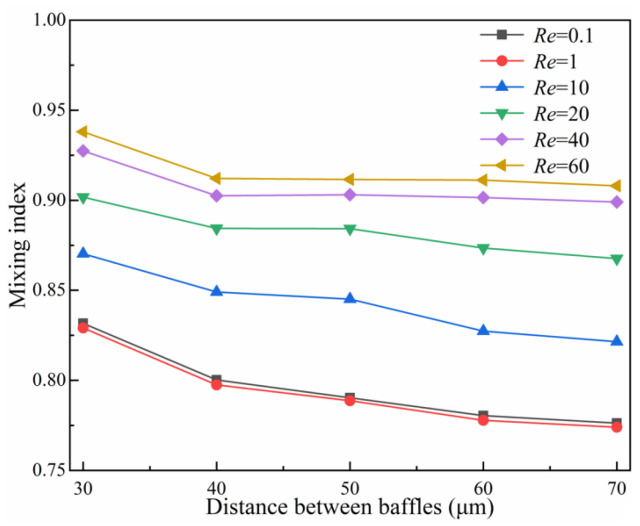
Effect of the distance of baffles on the mixed index.

**Figure 21:**
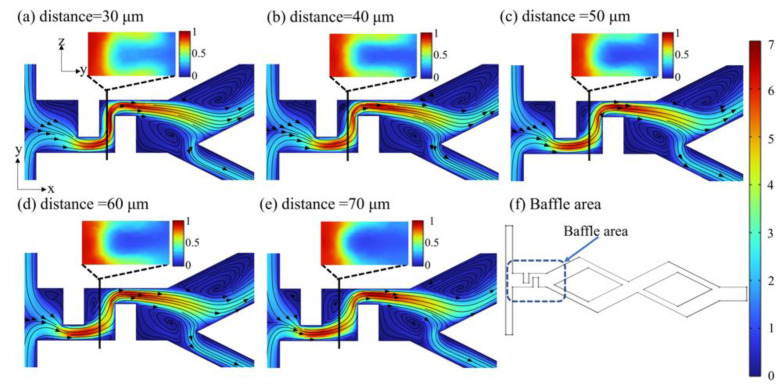
Velocity distribution (m/s) in baffle area and concentration distribution in baffle middle section at *Re* = 60. (**a**) distance = 30 μm; (**b**) distance = 40 μm; (**c**) distance = 50 μm; (**d**) distance = 60 μm; (**e**) distance = 70 μm; (**f**) Baffle area.

A second baffle with the same geometric parameters as baffle1 was used, and the position of the baffle1 was fixed in the optimal position 150 μm away from the inlet to explore the effect of the spacing between the baffle2 and the baffle1 (30 μm~70 μm) on the mixing efficiency. The mixing index curves under different baffle spacing at different *Re* are shown in Figure 20. The results show that the mixing index exhibits a downward trend with the increase in baffle spacing. The mixing index can achieve above 0.9 at *Re* = 40, 60; however, there is no significant change in the mixing efficiency with the baffle spacing gradually increasing from 40 μm~70 μm. Taking *Re* = 60 as an example, Figure 21 shows the velocity distribution in the baffle area and the concentration distribution in the middle section of the baffle. It can be found that with the decrease in the baffle spacing, the fluid flow through the baffle area is more rapid and receives stronger pipe resistance. Therefore, the baffles squeeze and fold the fluid more adequately, the boundary between the different fluids gradually becomes vaguer, and the fluid mixes more adequately. The optimal squeezing effect of the baffle on the fluid (Figure 21a) and the most adequate mixing of the fluid are observed at the baffle spacing of 30 μm.

## 6. Conclusions

In this paper, a novel asymmetric rhombus micromixer with a baffle is proposed to study the effect of multiple structural parameters on mixing. The flow field, concentration field, pressure drop and mixing index in the micromixer were analyzed numerically. Based on verifying the accuracy of the model, the multi-parameter influence rules of the rhombic angle, the width ratio of sub-channel and the size and relative positions of baffles, were discussed. The sensitivity of these mixing indexes to each structure was also studied.

The numerical calculation results indicate that the mixing mechanism is dominated by molecular diffusion in the range of *Re* = 0.1 and 1; the mixing index depends on the residence time of the fluid in the channel. Nonetheless, when *Re* = 10, 20, 40 and 60, the fluid convection plays a dominant position, and the channel structure becomes the main influencing factor in determining the mixing performance.

The comprehensive study of multiple structural parameters in this work indicates that the mixing index is not sensitive to the *cot θ*; The width ratio *w*_3_/*w*_4_ has an obvious influence on the mixing index. Due to the unbalanced collision of fluids, the mixing index will increase with the *w*_3_/*w*_4_, and then decrease slightly when it exceeds 6.5. The pressure drop is positively correlated with the *w*_3_/*w*_4_. Greater *w*_3_/*w*_4_ will lead to higher pressure drop in the channel, which is disadvantageous to the pump power and channel encapsulation. In addition, setting baffles in the micro-mixer can achieve a better mixing performance over the asymmetric design of microchannels. Among the baffles design parameters, the number of baffles and height of baffles have the most obvious influence on the mixing index. More baffles with higher heights stretch the fluid more strongly to increase its contact surface, facilitating mixing.

In this paper, the influence of multiple parameters on the mixing index is studied, and based on this, the micro-mixer is designed to achieve a mixing index greater than 0.9 in a wide range of *Re*, with good mixing performance, which provides a clear reference for the structure design of passive micro-mixer.

## Figures and Tables

**Table 1:** Structural parameters of rhombic separation and recombination micromixer.

Structure	Size (μm)
T-Channel entry width (*W*_1_, *W*_2_)	50
Mixed main inlet length (*D_in_*)	250
Mixed main inlet width (*W_in_*)	100
Mixing main outlet length (*D_out_*)	200
Mixed main outlet width (*W_out_*)	100
Hybrid wide channel (*w*_3_)	100
Hybrid narrow channel (*w*_4_)	40
Rhombus long semi-axis (*S*_1_)	250
Rhombus short half shaft (*S*_2_)	125
Rhombic angle (*θ*)	26.5°
Baffle width (*B*)	40
Baffle height (*H*)	70
Baffle position (*L_0_*)	150
Baffle spacing (*m*)	40
Micromixer height (*h*)	50
Micromixer length (*L*)	1769.6

**Table 2:** The calculated data of grid convergence index.

The Number of Grid Control Units (N_1_, N_2_, N_3_)			Mesh Refinement Rate (r)	Relative Error (ε)	Safety Factor (F_S_) [37]	The Order of Accuracy (p)	Grid Convergence Index (GCI)
100,054	251,766	400,477	2.5	−0.026	3	1.68	2.437

## Data Availability

The data are contained within the aiticle. To estimate the analysis results, the auther used the original data from the databass included in the references listed as [38].
